# Network meta-analysis of randomized controlled trials comparing the effectiveness of different treatments in reducing amniocentesis-associated pain and anxiety

**DOI:** 10.1186/s12884-023-06094-3

**Published:** 2023-11-21

**Authors:** Abdelrahman Mohamed Mahmoud, Abdallah R. Allam

**Affiliations:** Faculty of Medicine, Menoufia University, Yassin Abdelghaffar Street From Gamal Abdelnaser Street, Shebin Al-Kom, Menoufia Egypt

**Keywords:** Amniocentesis, Pain, Anxiety, Network meta-analysis

## Abstract

**Objective:**

This network meta-analysis compared different methods to determine which is most efficient at lowering pain and anxiety in women undergoing amniocentesis.

**Method:**

We looked through all published randomized controlled trials in the databases PubMed, Scopus, Web of Science, Cochrane, and EM base. Anxiety and pain were the predominant results. We used the R software version 4.2.1 to analyze the data.

**Results:**

We included a total of 20 studies, with sample sizes ranging from 60 to 570. Virtual reality was the most effective strategy for lowering pain during AC [MD = -1.30, 95% CI (-2.11, -0.49)]. In addition, paracetamol use was the most successful approach for lowering pain following AC [MD = -1.68, 95% CI (-1.99, -1.37)]. The use of H7 acupressure, however, was the strategy that significantly reduced anxiety following AC [SMD = -15.46, 95% CI (-17.77, -13.15)].

**Conclusion:**

The most effective method for reducing pain is the combination of virtual reality with paracetamol. Whereas, the most effective way to reduce anxiety is to combine an ice gel pack with H7 acupressure before applying AC.

**Supplementary Information:**

The online version contains supplementary material available at 10.1186/s12884-023-06094-3.

## Introduction

Prenatal diagnosis is an approach used by obstetricians to predict possible outcomes for each pregnancy such as congenital infections, alloimmunization, fetal genetic disorders, and fetal lung maturity. Receiving a prenatal diagnosis of congenital defects is momentous and emotionally challenging for women [1]. However, it provides a crucial opportunity for early intervention and informed decision-making regarding the management and care of the fetus [2].

The detection of fetal defects such as aneuploidy, a structural chromosome problem, requires invasive prenatal procedures such as amniocentesis (AC) and chorionic villus sampling (CVS) [1]. Over 190,000 AC procedures were carried out in the United States in 1997, making it a regularly used technique in obstetric practice [3]. The process is performed between 15 – 20 weeks after pregnancy, and the results are available in 7 to 14 days [4]. However, it carries risks such as membrane leakage, infection, and abortion with rates of 1.6%, 0.05%, and 1%, respectively [1, 5, 6]. Therefore, prenatal counseling is essential to prevent avoidable risks and costs for pregnant women [7].

High levels of anxiety have been reported in women undergoing AC [8], which may be due to fear of pain, fetal injury, and abortion, as well as concern about unfavorable results [9]. Moreover, anxiety might prolong the duration of the AC procedure, and contribute to procedure complications [10]. Studies have established a direct correlation between anxiety levels and pain intensity [11], with severe anxiety associated with increased pain during AC and chorionic villus sampling [11–14].

Even the smallest hint of pain could make the patient uncooperative and prevent a successful prenatal diagnosis. Therefore, several studies looked into various methods to help women getting AC to feel less pain and anxiety. While some of these studies found their method helpful [15–17], others did not [18–20].

Therefore, our network meta-analysis (NMA) aims to compare different pain and anxiety management strategies to identify the most effective approach for minimizing discomfort during AC procedures.

## Method

We followed the “Preferred Reporting Items for Systematic Reviews and Meta-Analyses (PRISMA)” standards to carry out this study [21]. Additionally, we strictly followed the steps provided in the “Cochrane Handbook for Systematic Reviews of Interventions” [22].

### Literature search strategy

From inception till August 2022, the following keywords were used to search PubMed, Scopus, Web of Science, and Cochrane Central: amniocentesis, amniocenteses, anesthesia, “local anesthesia”, lidocaine, “xylocaine”, EMLA, “lidocaine-prilocaine”, lignocaine, prilocaine, dalcaine, xylocitin, xylesthesin, xyloneural, otocaine, music, cryoanalgesia, “cold therapy”, “virtual reality”, “H7 acupressure”, “ethyl chloride”, “aromatic therapy”, “Light Pressure Effleurage”, education, paracetamol, Panadol, cryotherapy, “cold pack”, “Subfreezing room”, “Subfreezing”, “Light leg rubbing”, massage, analgesia, analgesic. Additionally, manual searches were conducted on Google Scholar, ResearchGate, and clinicaltrials.gov.

### Inclusion and exclusion criteria

We took included all randomized controlled trials (RCTs) that enrolled patients undergoing AC, compared various analgesics with one another, with a control, or with a placebo, and reported on pain perception, and anxiety. We excluded in vitro research, overlapping datasets, book chapters, reviews, cohort studies, case–control studies, and non-English articles. Using Endnote software, duplicates were eliminated, and then titles and abstracts of the retrieved references were checked. Eligible articles were then retrieved and underwent full-text screening. Additionally, we manually searched the reference lists of the papers that were included for other potentially qualifying studies.

### Data extraction

We extracted the summary data, the population's baseline demographics, and the efficacy outcomes from the included studies.

### Outcomes

Effective outcomes included anticipated pain, pain during the procedure, pain after the procedure, anxiety before the procedure, anxiety after the procedure, post-procedure pain, and anxiety, as well as willingness to undergo AC again if necessary.

### Risk of bias

We used the Cochrane risk of bias instrument (version 2) as described in chapter 8.5 of the Cochrane Handbook to evaluate the risk of bias [23]. The randomization process, deviations from intended interventions, missing outcome data, measurement of the outcome, selection of the reported result, and overall bias were part of the quality assessment process.

### Data analysis

We used the meta and netmeta tools in the R program version 4.2.1, to carry out network analysis. For pooling continuous outcomes, we used the mean difference (MD) or standardized mean difference (SMD), and for dichotomous outcomes, we used the risk ratio (RR), both with a 95% confidence interval (CI). Chi-square and I-square tests were used to determine how heterogeneous the pooled studies were, with a heterogeneous connection being defined as one where the I2 > 50% and the Chi-square *P*-value < 0.1. For pooling homogeneous data, we utilized a common effect model, and for heterogeneous data, we used random-effect model.

## Results

### Data collection and characteristics of included studies

**Fig. 1:**
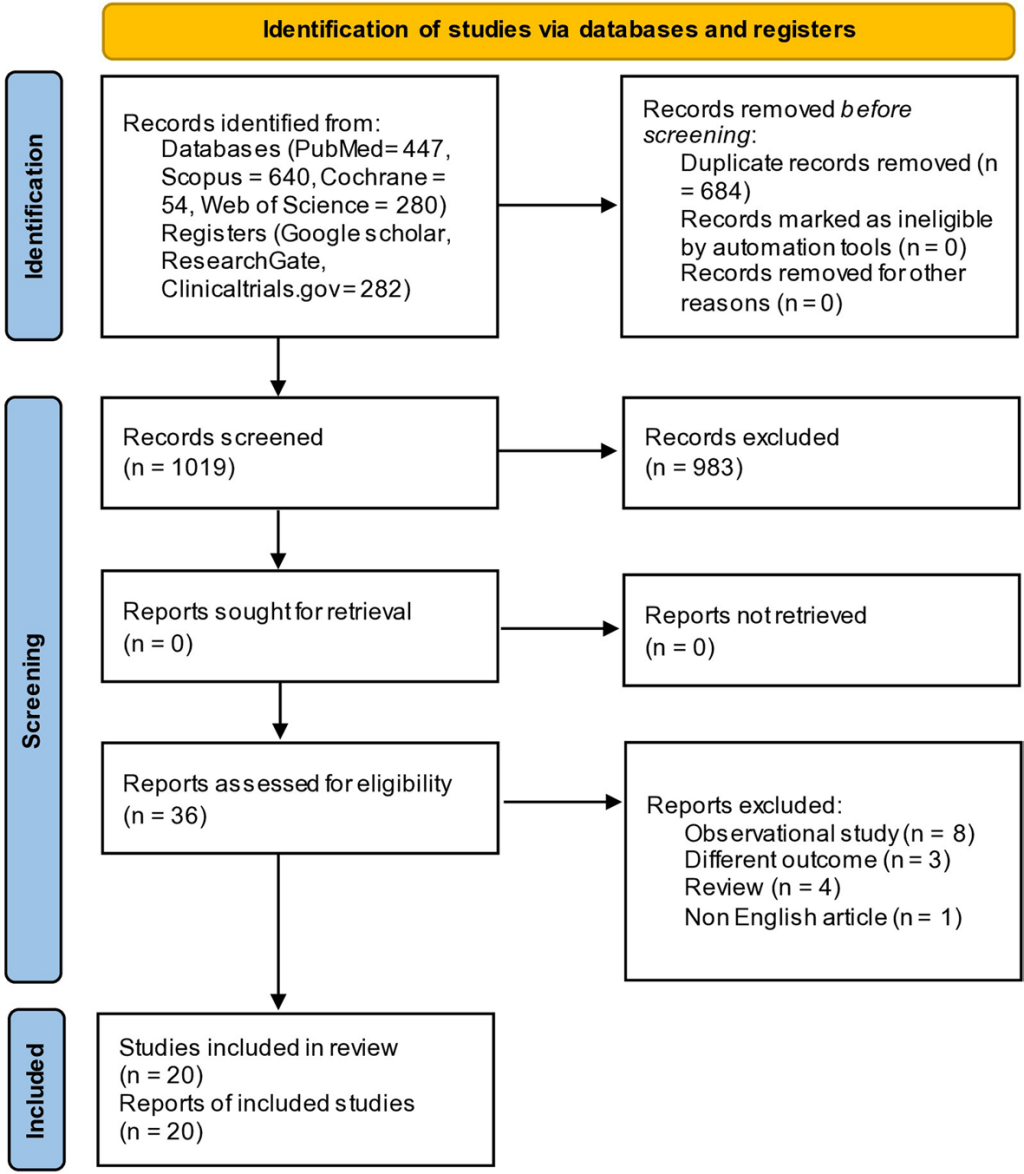
Preferred reporting item for systematic review and meta-analysis

One thousand seven hundred three articles were found during database searches: 447 from PubMed, 280 from Web of Science, 640 from Scopus, 54 from CENTRAL, and 282 from other databases. We filtered 1019 items and deleted 684 duplicates. 983 were eliminated through the title and abstract screening process, and 20 [15–20, 24–37] acceptable studies were found after the full-text screening of the 36 publications that remained. A flowchart of the database search and study selection procedure is shown in Fig. 1.

The sample sizes of the included RCTs ranged from 60 to 570 people. Different approaches were contrasted with one another, with a control group, or with a placebo. The included subjects' average age ranged from 31.3 to 37.7 years, their average gestational age was 15.9 to 21.95 weeks, and their average body mass index was 22.93 to 27 kg/m^2^. The summary and the baseline characteristics of the included subjects in each study are shown in Tables 1 and 2.

**Table 1:** Summary of the included studies

Study ID	Study Design	NCT number	Site	Recruitment duration	Total Participants	Intervention	Control
Melcer 2021 [15]	Open Label RCT	NCT04491149	Israel	September to October 2020	65	VR (35)	Control (30)
Mohammadifard 2021 [16]	Single-blind RCT	IRCT20200401046914N1	Iran	June to September 2020	60	H7_Acupressure (30)	Control (30)
Rekawek 2019 [18]	Open Label RCT	NCT03140293	USA	October 2016 to May 2017	120	lidocaine_injection (63)	Ethyl_chloride (57)
Pongrojpaw 2007 [19]	Double-blind RCT	-	Thailand	October 2006 to April 2007	120	Lidocaine_prilocaine_cream (60)	Placebo (60)
Tuaktaew 2018 [17]	Double-blind RCT	NCT03035045	Thailand	August 2016 to June 2017	240	Paracetamol (120)	Placebo (120)
Schoubroeck 2000 [24]	Open Label RCT	-	Belgium	April to November 1998	220	lidocaine_injection (114)	Control (106)
Wax 2005 [25]	Single-blind RCT	-	USA	-	62	Subfreezing_temperature_needle (29)	Room_temperature_needle (33)
Telapol 2018 [26]	Open Label RCT	-	Thailand	May to November 2016	148	Ethyl_chloride (74)	Control (74)
Benchahong 2021 [27]	Single-blind RCT	TCTR20191115002	Thailand	December 2019 to May 2020	480	Ice_gel_pack_before_AC (120), Ice_gel_pack_after_AC (120), Ice_gel_pack_before_after_AC (120)	Control (120)
Elimian 2013 [28]	Double-blind RCT	NCT 00583011	USA	October 2007 to September 2009	76	lidocaine_injection (36)	Control (40)
Fischer 2000 [29]	Single-blind RCT	-	USA	April 1998 to July 1999	200	Effleurage (103)	Control (97)
Gordon 2007 [30]	Single-blind RCT	-	USA	January 1995 to March 2001	204	lidocaine_injection (101)	Control (103)
Hanprasertpong 2012 [31]	Single-blind RCT	-	Thailand	July 2009 to July 2010	372	Ice_gel_pack_before_AC (184)	Control (188)
Hanprasertpong 2015 [32]	Single-blind RCT	-	Thailand	July to September 2013	317	Menthol (158)	Control (159)
Hanprasertpong 2016 [20]	Single-blind RCT	-	Thailand	February to May 2013	332	Music (161)	Control (171)
Katsogiannou 2018 [33]	Single-blind RCT	-	France	March 2013 to February 2015	183	Nitrous_oxide (93)	Control (90)
Kuemanee 2021 [34]	Single-blind RCT	TCTR20191116001	Thailand	December 2019 to March 2020	240	Ice_gel_pack_before_AC (120)	Control (120)
Homkrun 2019 [35]	Single-blind RCT	TCTR20170528001	Thailand	June 2017 to January 2018	570	Xylocaine (191)	Control (379)
Kang 2020 [36]	Single-blind RCT	-	China	June 2012 to June 2014	100	Psychological_intervention (48)	Control (52)
Mojahed 2021 [37]	Open Label RCT	-	Iran	2020	80	Education (40)	Control (40)

Study ID	Inclusion criteria	Exclusion criteria	Ascertainment of pain	Aim
Melcer 2021 [15]	The trial was open to consecutive women who were going to get an obstetrically indicated mid-trimester AC	Women under the age of eighteen or with numerous pregnancies were not included in the sample. Women who needed pre-procedural anxiolytic or analgesic medication, had a history of motion sickness, epilepsy, or had a hearing or visual impairment were also eliminated	VAS	The study's objective is to compare the effectiveness of a VR intervention to a control group in terms of controlling acute pain and anxiety during amniocentesis
Mohammadifard 2021 [16]	The following conditions had to be met in order to qualify: gestational age of 15 to 18 weeks, literacy, desired pregnancy, score of ≤ 53 on the Spielberger Anxiety Inventory, absence of any obstetrical issues or medical conditions, abstinence from drugs and alcohol, absence of abnormal hand findings, maternal BMI in the range of 18.5 to 30 and absence of any history of recurrent abortions (more than three consecutive abortions), amniocentesis, known mental	Due to their unwillingness to cooperate, active vaginal bleeding, hospitalization, failure to complete the intervention twice weekly or three times intermittently throughout the study, multiple failed attempts at amniocentesis, and unusual sensitivity to pressure point touch, the participants were excluded from the study	-	The purpose of this study was to find out how H7 Acupressure affected pregnant women's anxiety during amniocentesis procedures
Rekawek 2019 [18]	The trial was open to all singleton pregnant women who underwent transabdominal CVS between 10 and 13 weeks and 6 days of gestation	Multiple pregnancies, known medication allergies, and hypersensitivity to local anesthetic were among the exclusion criteria	VAS	Therefore, the purpose of this study was to ascertain if topical ethyl chloride anesthetic spray administration compared to 1% lidocaine subdermal injection results in lower pain perception during transabdominal CVS
Pongrojpaw 2007 [19]	All of the expectant women who took part in the current study gave their consent to have an AC at the Maternal–Fetal Medicine Unit and were referred for genetic counseling in the second trimester of pregnancy	Multiple pregnancies or severe congenital anomalies found by sonography were excluded, as were known or suspected allergies to lidocaine prilocaine, psychiatric disorders, multiple attempts to insert a needle, and switching the puncture site where the cream was given because of fetal activity	VAS	The current study set out to determine whether local lidocaine-prilocaine cream application did in fact lessen pain during mid-trimester genetic AC
Tuaktaew 2018 [17]	For this study, the singleton pregnant patients undergoing their first genetic AC at Rajavithi Hospital's Department of Obstetrics and Gynecology were considered eligible	Psychiatric disorders, paracetamol allergies, AC in cases of suspected fetal anomalies identified by another prenatal diagnostic procedure, a patient's history of paracetamol use in the 24–48 h prior to the amniocentesis, and participants who declined to enroll in the study were the exclusion criteria	VAS	The study's goal is to determine whether paracetamol can reduce pain scores during AC
Schoubroeck 2000 [24]	Pregnant women undergoing AC	Multiple pregnancies, a known or suspected lignocaine allergy, an AC performed right away following the sonographic discovery of a severe fetal abnormality, a psychiatric illness, and a lack of proficiency in Dutch are all factors	VAS and VRS	This study sought to determine whether local anesthesia reduced pain during AC
Wax 2005 [25]	Participants in this institutionally approved study had to be females aged ≥ 18 who underwent an indicated second trimester genetic amniocentesis, be carrying a singleton, have a normal amniotic fluid volume, and not have had an amniocentesis or chorionic villus sampling (CVS) in the previous pregnancy	Women who had previously undergone AC or CVS in the current pregnancy	VAS	In order to lessen pain associated with second trimester genetic AC, we conducted a randomized single-blinded trial comparing needles that were frozen to those that were at room temperature
Telapol 2018 [26]	Women who had never undergone AC and no fetal gross structural abnormalities detected by ultrasonographic examination were the inclusion criteria	Women who were known to be allergic to colds, ethyl chloride spray, had taken painkillers within the previous four hours, needed more than one puncture during the same procedure, could not follow the study's methodology, or had poor communication skills, were all excluded from the study	VAS	This study's goal was to evaluate the cryo-analgesic impact of ethyl chloride spray on pain management during AC in the second trimester
Benchahong 2021 [27]	Pregnancy, being between the ages of 18 and 45, having between 15 and 20 weeks of gestation, being without any ultrasonographic signs of foetal anomalies, and choosing AC as the prenatal diagnosis procedure are the inclusion criteria	Multifetal pregnancy, ultrasound evidence of a severe congenital anomaly, altered puncture site following cold compression during AC, repeated attempts, history of cold urticaria, pregnancy with Raynaud's phenomenon, use of painkillers that impair pain and temperature perception, abdominal skin infection, pregnancy with psychosis, and patients' refusal are all exclusion criteria	VAS	Evaluating the impact of cold therapy on a patient's perceived pain levels before and after an AC surgery
Elimian 2013 [28]	Ages between 18 and 45, consent for participation, singleton pregnancies, and gestational ages between 15 and 23 weeks were all inclusion criteria	We disqualified multiple-gestational women, people taking painkillers or other analgesics, people who declined to participate, and people who have a known lidocaine hypersensitivity. Additionally, we didn't include cases in which amnioinfusion or amnioreduction were advised	VAS and NRS	Assessing how local anaesthetic affects how much pain the mother feels during AC
Fischer 2000 [29]	-	-	VAS	Determining whether leg rubbing with gentle pressure during genetic AC lessens pain and anxiety associated with the procedure
Gordon 2007 [30]	-	-	VAS and NRS	The null hypothesis states that local anaesthetic does not reduce AC patient pain perception
Hanprasertpong 2012 [31]	Women who had second-trimester genetic AC due to advanced maternal age who were between 15 and 21 weeks pregnant (according to the latest menstrual period or ultrasonographic-biometric measurement)	Multiple pregnancies, a history of AC during a prior or ongoing pregnancy, the presence of foetal structural malformation, more than one attempt at needle insertion, participants who were unable to read or understand the questionnaire, or participants who declined to participate in our study were all exclusion criteria	VAS	Determining whether cryoanalgesia reduces the level of pain experienced during genetic AC in the second trimester
Hanprasertpong 2015 [32]	Expectant women scheduled for genetic AC between 15 and 20 weeks of gestation because of advanced maternal age	A foetal structural malformation, multiple pregnancies, AC experience during the current or a previous pregnancy, multiple needle insertion attempts, a history of smell or taste perception issues, a history of an upper respiratory infection or a diagnosis of allergic rhinitis within two weeks of the procedure, an inability to read or understand the questionnaire, and a refusal to participate in the study were all grounds for exclusion	VAS	Assessing the effectiveness of menthol-based aromatic treatment to reduce AC-related pain
Hanprasertpong 2016 [20]	Women who had a second trimester genetic AC due to advanced maternal age and were between 15 and 21 weeks pregnant based on their previous period or an ultrasonographic biometric measurement	Multiple pregnancies, foetal structural malformations, history of AC in a prior or ongoing pregnancy, multiple attempts at needle insertion, a history of hearing impairment, and participants who were illiterate, incapable of understanding the questionnaires, or who refused to participate in the study were all disqualified	VAS	Determining whether listening to music during genetic AC in the second trimester reduced pain perception
Katsogiannou 2018 [33]	Patients had to be pregnant adults (over 18), have gestational ages between 11 and 16 weeks, be receiving transabdominal CVS, have no contraindications to using N_2_O or local anaesthetic, and have no contraindications to transabdominal CVS	-	VAS	Our goal was to assess how well nitrous gas and local anaesthetic managed pain and anxiety during transabdominal CVS
Kuemanee 2021 [34]	The present study included pregnant women who had genetic AC between 15 and 22 weeks of gestation	Multifetal pregnancy, severe congenital anomaly previously detected by ultrasonography, cases of multiple needle puncture attempts during the procedure, cases of changing the puncture site due to foetal behavior, maternal psychiatric disorder, those who were contraindicated to cold therapy, and cases of refusal to participate in the current study were excluded	VAS	Assessing the impact of cryotherapy on pain management during genetic AC in the second trimester
Homkrun 2019 [35]	Singleton pregnancy and gestational ages of 16 to 20 weeks based on a trustworthy last menstrual cycle and sonographic biometry in the first half of pregnancy were the inclusion criteria	Pregnant women who (1) had a history of Xylocaine allergy, (2) had aberrant sensory function based on history, (3) couldn’t rate their pain using a visual analogue scale, and (4) couldn’t do an AC were excluded	VAS	Evaluating the impact of Xylocaine spray on the AC pain score
Kang 2020 [36]	-	-	VAS	The purpose of this study is to investigate how psychological psychotherapy can reduce pregnancy-related anxiety and dread in pregnant women as well as the surgical success rate
Mojahed 2021 [37]	Muslim and Iranian ethnicity, written informed consent to participate in the study, a minimal level of literacy, AC eligibility, gestational age of 15–20 weeks, a single pregnancy with a viable fetus, and a positive foetal screening test were the inclusion criteria	The study's initial exclusion criteria included pregnancy after infertility treatment and assisted reproductive techniques, history of AC, history of recurrent miscarriages, presence of major abnormalities in ultrasound, awareness of the specifics of amniocentesis, use of hookah, cigarettes, drugs, alcohol, psychotropic drugs, history of consulting a psychiatrist or psychologist for mood and mental disorders, taking medication, or hospitalization	-	The purpose of this study was to ascertain how schooling affected moms who were amniocentesis candidates' perceptions of stress

*Abbreviations*: *RCT* Randomized controlled trial, *VR* Virtual reality, *BMI* Body mass index, *VAS* Visual analogue scale, *NRS* Numerical rating scale, *AC* Amniocentesis, *CVS* Chorionic villous sampling, *N*_*2*_*O* Nitrous oxide

**Table 2:** Baseline characteristics of included participants

Study ID	Type of intervention	Number of participants in each group		Age (Years), M (SD)		Weight (Kg), M (SD)		Height (m), M (SD)		BMI (Kg/m^2^), M(SD)	
		**Intervention**	**Control**	**Intervention**	**Control**	**Intervention**	**Control**	**Intervention**	**Control**	**Intervention**	**Control**
Melcer 2021 [15]	VR	30	30	34.9 (4.9)	36.7 (3.3)	-	-	-	-	-	-
Mohammadifard 2021 [16]	H7_Acupressure	27	29	-	-	-	-	-	-	-	-
Rekawek 2019 [18]	Lidocaine_injection	63	57	36.14 (4.87)	35.6 (4.91)	-	-	-	-	24.38 (4.82)	24.22 (5.34)
Pongrojpaw 2007 [19]	Lidocaine_prilocaine_cream	60	60	36.8 (3.79)	36.9 (3.41)	-	-	-	-	24.4 (4.2)	24.1 (3.6)
Tuaktaew 2018 [17]	Paracetamol	117	116	36.26 (3.78)	36.01 (4.66)	-	-	-	-	24.93 (3.81)	24.99 (4.98)
Schoubroeck 2000 [24]	Lidocaine_injection	114	106	34.1 (3.9)	33 (4.6)	62.7 (12.1)	67.7 (13)	-	-	-	-
Wax 2005 [25]	Subfreezing_temperature_needle	29	33	34.4 (6.1)	35.6 (3.6)	69 (15.3)	74.7 (18.1)	1.667 (0.071)	1.651 (0.071)	-	-
Telapol 2018 [26]	Ethyl_chloride	74	74	37.80 (2.83)	37.35 (2.54)	60.01 (10.59)	60.75 (10.34)	1.57 (0.0524)	1.59 (0.0605)	24.34 (4.07)	24.11 (3.82)
Benchahong 2021 [27]	Ice_gel_pack_before_AC	120	120	36.85 (3.07)	36.48 (3.71)	62 (10.07)	61.75 (10.73)	1.57 (5.8)	1.58 (5.47)	24.87 (3.61)	24.73 (4.16)
	Ice_gel_pack_after_AC	120		36.8 (3.72)		62.36 (11.28)		1.58 (5.8)		24.6 (4.27)	
	Ice_gel_pack_before_after_AC	120		37.07 (3.07)		60.4 (11.28)		1.57 (5.47)		24.26 (4.38)	
Elimian 2013 [28]	Lidocaine_injection	36	40	31.3 (6.5)	30.1 (7.5)	75.8 (14)	77.2 (17.1)	1.65 (0.06)	1.66 (0.08)	-	-
Fischer 2000 [29]	Effleurage	103	97	33.3 (5.2)	34.4 (5.3)	71.2 (13.6)	71.1 (17)	-	-	27 (5.1)	26 (5.8)
Gordon 2007 [30]	Lidocaine_injection	101	103	33.7 (5.7)	33.3 (5.9)	71.4 (11.3)	72.5 (15.4)	1.63 (0.07)	1.62 (0.07)	26.4 (3.8)	27.3 (5.1)
Hanprasertpong 2012 [31]	Ice_gel_pack_before_AC	184	188	37.3 (2.51)	37.1 (2.47)	-	-	-	-	24.3 (3.8)	24.4 (4.12)
Hanprasertpong 2015 [32]	Menthol	158	159	37.1 (2.1)	37.54 (2.57)	-	-	-	-	24.1 (4)	23.7 (3.5)
Hanprasertpong 2016 [20]	Music	161	171	37.4 (2.5)	37.1 (2.37)	-	-	-	-	25.27 (4.33)	24.8 (4.26)
Katsogiannou 2018 [33]	Nitrous_oxide	93	90	37.7 (0.54)	34.27 (0.64)	-	-	-	-	24.23 (0.57)	22.93 (0.5)
Kuemanee 2021 [34]	Ice_gel_pack_before_AC	120	120	36.8 (3.7)	36.5 (3.9)	-	-	-	-	24.9 (4.2)	24.7 (4.1)
Homkrun 2019 [35]	Xylocaine	191	379	36 (3)	36 (3)	-	-	-	-	-	-
Kang 2020 [36]	Psychological_intervention	48	52	-	-	-	-	-	-	-	-
Mojahed 2021 [37]	Education	40	40	32.9 (5.85)	34 (5.5)	-	-	-	-	-	-

Study ID	Occupation, n (%)										Education, n (%)							
	**Housewife**		**Business owner**		**Government officer**		**Agriculture**		**Other**		**Less than high school**		**high school**		**Bachelor’s degree (collage)**		**Higher than bachelor’s degree**	
	**Intervention**	**Control**	**Intervention**	**Control**	**Intervention**	**Control**	**Intervention**	**Control**	**Intervention**	**Control**	**Intervention**	**Control**	**Intervention**	**Control**	**Intervention**	**Control**	**Intervention**	**Control**
Melcer 2021 [15]	-	-	-	-	-	-	-	-	-	-	-	-	-	-	-	-	-	-
Mohammadifard 2021 [16]	22 (81.5%)	25 (86.2%)	-	-	-	-	-	-	5 (18.5%)	4 (13.7%)	8 (29.6%)	4 (13.8%)	7 (25.9%)	14 (48.3%)	12 (44.4%)	11 (37.9%)	-	-
Rekawek 2019 [18]	-	-	-	-	-	-	-	-	-	-	-	-	5 (7.9%)	4 (7.4%)	46 (73%)	40 (74.1%)	12 (19.1%)	10 (18.5%)
Pongrojpaw 2007 [19]	-	-	-	-	-	-	-	-	-	-	-	-	-	-	-	-	-	-
Tuaktaew 2018 [17]	-	-	-	-	-	-	-	-	-	-	-	-	-	-	-	-	-	-
Schoubroeck 2000 [24]	-	-	-	-	-	-	-	-	-	-	-	-	-	-	-	-	-	-
Wax 2005 [25]	-	-	-	-	-	-	-	-	-	-	-	-	-	-	-	-	-	-
Telapol 2018 [26]	-	-	-	-	-	-	-	-	-	-	-	-	-	-	-	-	-	-
Benchahong 2021 [27]	16 (13.3%)	9 (7.5%)	21 (17.5%)	15 (12.5%)	13 (10.8%)	19 (15.8%)	-	-	70 (58.3%)	77 (64.2%)	26 (21.7%)	30 (25%)	37 (30.8%)	38 (31.7%)	50 (41.7%)	40 (33.3%)	7 (5.8%)	12 (10%)
	11 (9.2%)		18 (15%)		14 (11.7%)		-	-	77 (64.2%)		27 (22.5%)		37 (30.8%)		49 (40.8%)		7 (5.8%)	
	10 (8.3%)		15 (12.5%)		12 (10%)		-	-	83 (69.2%)		29 (24.2%)		35 (29.2%)		50 (41.7%)		6 (5%)	
Elimian 2013 [28]	-	-	-	-	-	-	-	-	-	-	4 (11.11%)	5 (12.5%)	14 (38.89%)	17 (42.5%)	18 (50%)	17 (42.5%)	-	-
Fischer 2000 [29]	-	-	-	-	-	-	-	-	-	-	-	-	-	-	-	-	-	-
Gordon 2007 [30]	-	-	-	-	-	-	-	-	-	-	-	-	-	-	-	-	-	-
Hanprasertpong 2012 [31]	-	-	-	-	-	-	-	-	-	-	-	-	-	-	-	-	-	-
Hanprasertpong 2015 [32]	26 (16.46%)	23 (14.47%)	-	-	42 (26.58%)	38 (23.90%)	13 (8.23%)	12 (7.55%)	77 (48.73%)	86 (54.09%)	-	-	59 (37.34%)	62 (38.99%)	98 (62.03%)	94 (59.12%)	1 (0.63%)	3 (1.89%)
Hanprasertpong 2016 [20]	28 (17.39%)	34 (19.88%)	-	-	40 (24.84%)	44 (25.73%)	13 (8.07%)	17 (9.94%)	80 (49.69%)	76 (44.44%)	-	-	61 (37.89%)	70 (40.49%)	97 (60.25%)	101 (59.06%)	3 (1.86%)	0 (0%)
Katsogiannou 2018 [33]	-	-	-	-	-	-	-	-	-	-	-	-	-	-	-	-	-	-
Kuemanee 2021 [34]	-	-	18 (15%)	15 (12.6%)	14 (11.7%)	19 (15.8%)	-	-	11 (9.2%)	9 (7.5%)	-	-	-	-	-	-	7 (5.7%)	12 (10%)
Homkrun 2019 [35]	-	-	-	-	-	-	-	-	-	-	-	-	-	-	-	-	-	-
Kang 2020 [36]	-	-	-	-	-	-	-	-	-	-	-	-	-	-	-	-	-	-
Mojahed 2021 [37]	-	-	-	-	-	-	-	-	-	-	-	-	-	-	-	-	-	-

Study ID	Previous abdominal surgery, n (%)		Gestational age at AC or CVS (weeks), M(SD)		Gravidity, M(SD)		Parity, M (SD)		Parity, n (%)			
									**Nulliparous**		**Multiparous**	
	**Intervention**	**Control**	**Intervention**	**Control**	**Intervention**	**Control**	**Intervention**	**Control**	**Intervention**	**Control**	**Intervention**	**Control**
Melcer 2021 [15]	-	-	18.9 (1.3)	18.4 (0.6)	2.4 (1.4)	3.1 (1.9)	0.9 (0.9)	1.3 (1.3)	-	-	-	-
Mohammadifard 2021 [16]	-	-	-	-	-	-	-	-	2 (7.4%)	8 (27.6%)	25 (92.6%)	21 (72.4%)
Rekawek 2019 [18]	22 (34.9%)	12 (21.1%)	-	-	-	-	-	-	22 (34.9%)	26 (45.6%)	41 (65.1%)	31 (54.4%)
Pongrojpaw 2007 [19]	20 (33%)	12 (20%)	17.6 (1.6)	17.7 (1.5)	-	-	0.7 (0.8)	0.6 (0.7)	-	-	-	-
Tuaktaew 2018 [17]	-	-	18.43 (0.82)	18.40 (0.7)	-	-	-	-	-	-	-	-
Schoubroeck 2000 [24]	-	-	15.9 (1.3)	15.8 (1.4)	2.4 (1.2)	2.6 (1.4)	1.1 (0.9)	1.1 (1.1)	-	-	-	-
Wax 2005 [25]	-	-	16.5 (1.4)	16.6 (1.5)	-	-	-	-	-	-	-	-
Telapol 2018 [26]	10 (13.51%)	8 (10.81%)	21.95 (0.88)	22.02 (0.80)	-	-	-	-	22 (29.73%)	27 (36.49%)	52 (70.27%)	47 (63.51%)
Benchahong 2021 [27]	-	-	-	-	-	-	-	-	-	-	-	-
	-	-	-	-	-	-	-	-	-	-	-	-
	-	-	-	-	-	-	-	-	-	-	-	-
Elimian 2013 [28]	-	-	19.8 (2.6)	20.1 (2.4)	-	-	-	-	7 (19.44%)	12 (30%)	29 (80.55%)	28 (70%)
Fischer 2000 [29]	-	-	17.5 (1.5)	17 (1.4)	2.4 (0.8)	2.5 (0.7)	-	-	32 (31.1%)	25 (25.8%)	71 (68.9%)	72 (74.2%)
Gordon 2007 [30]	40 (39.6%)	44 (42.7%)	19.3 (5.5)	19.9 (6.6)	2.8 (1.4)	2.7 (1.6)	1.2 (1)	1.2 (1.1)	-	-	-	-
Hanprasertpong 2012 [31]	78 (42.4%)	73 (38.8%)	17.09 (0.89)	17.02 (0.76)	-	-	-	-	-	-	-	-
Hanprasertpong 2015 [32]	73 (46.2%)	86 (54.09%)	-	-	-	-	-	-	-	-	-	-
Hanprasertpong 2016 [20]	79 (49.07%)	77 (45.03%)	17.27 (0.86)	17.23 (0.83)	-	-	-	-	-	-	-	-
Katsogiannou 2018 [33]	-	-	13.83 (0.09)	13.91 (0.09)	-	-	-	-	40 (43%)	29 (32.2%)	53 (57%)	61 (67.8%)
Kuemanee 2021 [34]	30 (25%)	40 (30.33%)	-	-	-	-	0.8 (0.67)	0.8 (0.83)	-	-	-	-
Homkrun 2019 [35]	-	-	17 (1)	17 (2)	-	-	-	-	46 (24.08%)	101 (26.64%)	145 (75.9%)	278 (73.35%)
Kang 2020 [36]	-	-	-	-	-	-	-	-	-	-	-	-
Mojahed 2021 [37]	-	-	16.35 (0.93)	16.5 (1.08)	-	-	-	-	-	-	-	-

Study ID	Indication, n (%)				Prior AC or CVS, n (%)		Prior pregnancy, n (%)		Prior abortion, n (%)	
	**Age risk**		**Genetic risk**							
	**Intervention**	**Control**	**Intervention**	**Control**	**Intervention**	**Control**	**Intervention**	**Control**	**Intervention**	**Control**
Melcer 2021 [15]	19 (63.3%)	23 (76.7)	-	-	3 (10%)	5 (16.7%)	-	-	12	12
Mohammadifard 2021 [16]	-	-	-	-	-	-	29 (100%)	27 (100%)	7 (25.9%)	7 (24.1%)
Rekawek 2019 [18]	36 (61%)	28 (52.8%)	-	-	25 (39.7%)	20 (35.7%)	-	-	-	-
Pongrojpaw 2007 [19]	56 (93%)	57 (95%)	4 (7%)	3 (5%)	1 (0.1%)	3 (0.5%)	-	-	-	-
Tuaktaew 2018 [17]	-	-	-	-	-	-	-	-	-	-
Schoubroeck 2000 [24]	54 (47%)	41 (39%)	2 (2%)	4 (4%)	-	-	-	-	-	-
Wax 2005 [25]	21 (69.69%)	23 (79.3%)	-	-	-	-	-	-	-	-
Telapol 2018 [26]	-	-	-	-	-	-	44 (59.46%)	41 (55.4%)	24 (32.43%)	20 (27.03%)
Benchahong 2021 [27]	109 (90.8%)	105 (87.5%)	11 (9.2%)	15 (12.5%)	-	-	-	-	-	-
	109 (90.8%)		11 (9.2%)		-	-	-	-	-	-
	105 (87.5%)		15 (12.5%)		-	-	-	-	-	-
Elimian 2013 [28]	-	-	-	-	-	-	-	-	-	-
Fischer 2000 [29]	61 (59.2%)	59 (60.8%)	-	-	12 (11.8%)	22 (22.7%)	-	-	-	-
Gordon 2007 [30]	-	-	-	-	-	-	-	-	-	-
Hanprasertpong 2012 [31]	-	-	-	-	-	-	-	-	-	-
Hanprasertpong 2015 [32]	-	-	-	-	-	-	-	-	-	-
Hanprasertpong 2016 [20]	-	-	-	-	-	-	-	-	-	-
Katsogiannou 2018 [33]	-	-	-	-	11 (11.8%)	8 (8.9%)	-	-	-	-
Kuemanee 2021 [34]	109 (90.9%)	105 (87.6%)	-	-	-	-	-	-	-	-
Homkrun 2019 [35]	-	-	-	-	-	-	-	-	-	-
Kang 2020 [36]	-	-	-	-	-	-	-	-	-	-
Mojahed 2021 [37]	-	-	-	-	-	-	-	-	-	-

*Abbreviations*: *VR* Virtual reality, *BMI* Body mass index, *M* Mean, *SD* Standard deviation, *m* meter, *n* number

### Risk of bias assessment

High to low-quality studies were included. The majority of the examined studies exhibited a high risk of bias across the board. In Supplementary Materials, Figs. S1 and S2 depict a risk of bias graph and a risk of bias summary, respectively.

### Outcomes

#### Anticipated pain (See Supplementary materials)

**Fig. 2:**
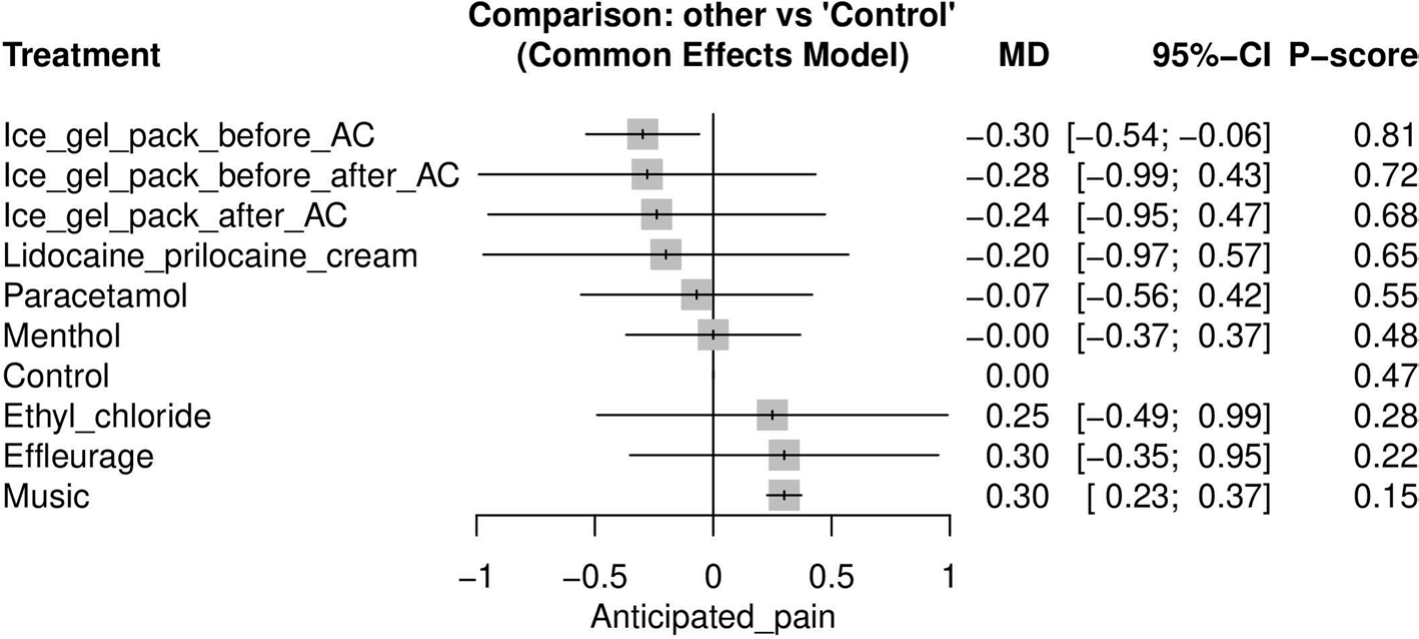
Network meta-analysis forest plot of anticipated pain

Anticipated pain was reported by 10 studies [17, 19, 20, 25–27, 29, 31, 32, 34]. However, we removed WAX et al. [25], because their study resulted in two isolated networks. The most efficient method for reducing anticipated pain when compared to control was using an ice gel pack before AC; this method was significantly better [MD = -0.30, 95% CI (-0.54, -0.06)]. Figure 2 displays the NMA forest plot.

#### Pain during AC (See Supplementary materials)

**Fig. 3:**
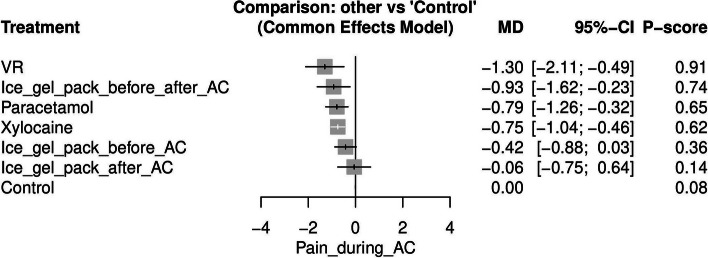
Network meta-analysis forest plot of pain during amniocentesis

Pain during AC was reported by six studies [15, 17, 18, 27, 34, 35]. However, we excluded Rekawek et al. [18], because their study resulted in two isolated networks. Virtual reality (VR) was the most effective approach for lowering pain during AC when compared to control [MD = -1.30, 95% CI (-2.11, -0.49)] after which ice gel pack before and after AC was the next most effective [MD = -0.93, 95% CI (-1.62, -0.23)]. Figure 3 displays the NMA forest plot.

#### Pain after AC (See Supplementary materials)

**Fig. 4:**
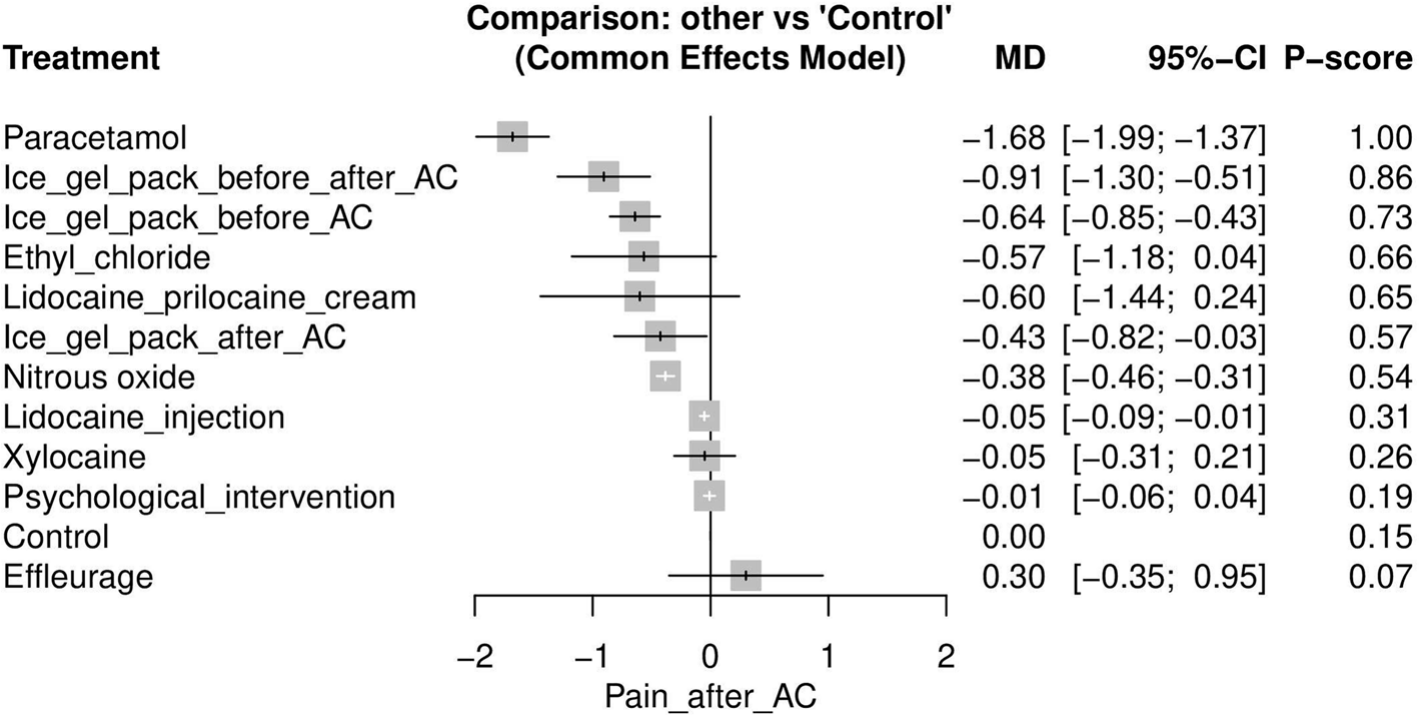
Network meta-analysis forest plot of pain after amniocentesis

Pain after AC was reported by 14 studies [17–19, 24–30, 33–36]. However, WAX et al. [25], was excluded because their study resulted in two isolated networks. Paracetamol was the most effective approach for lowering pain after AC when compared to control [MD = -1.68, 95% CI (-1.99, -1.37)] after which ice gel pack before and after AC was the next most effective [MD = -0.91, 95% CI (-1.30, -0.51)] Fig. 4 displays the NMA forest plot.

#### Anxiety before AC (See Supplementary materials)

**Fig. 5:**
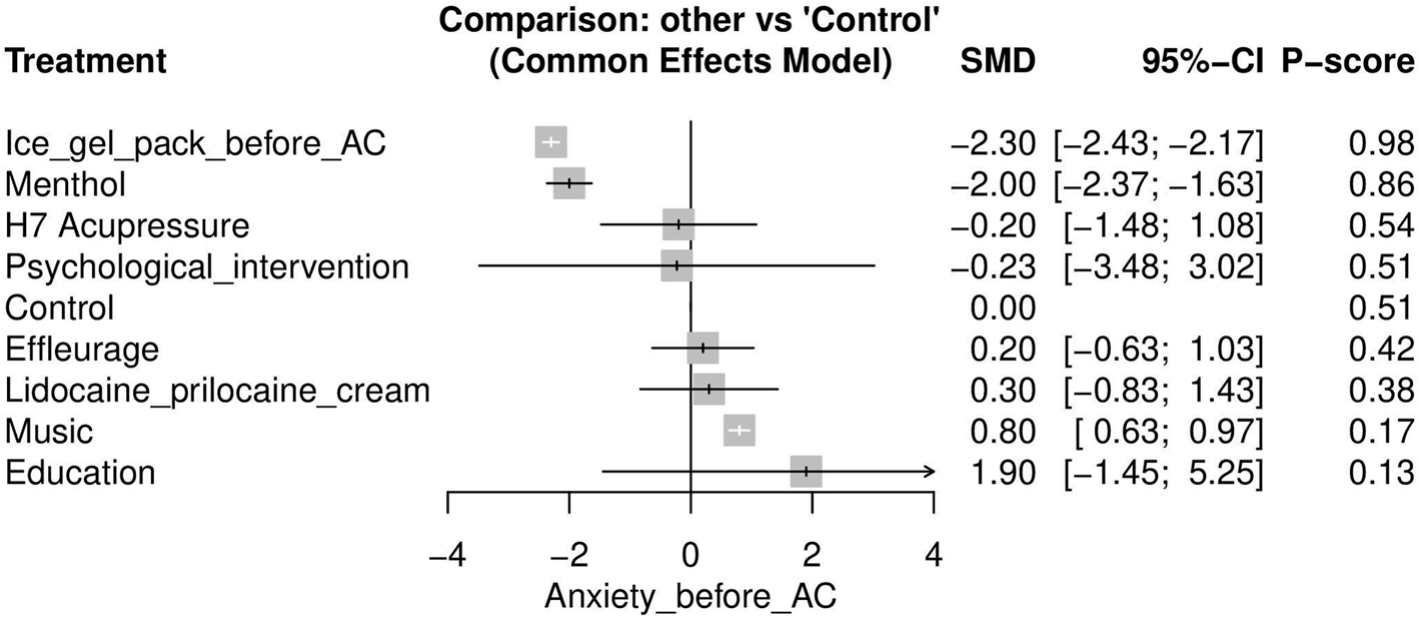
Network meta-analysis forest plot of anxiety before amniocentesis

Eight studies reported anxiety before AC outcome [16, 19, 20, 29, 31, 32, 36, 37] Ice gel pack before AC was the most effective approach for lowering anxiety before AC when compared to control [SMD = -2.30, 95% CI (-2.43, -2.17)] after which menthol was the next most effective [SMD = -2.00, 95% CI (-2.37, -1.63)]. Figure 5 displays the NMA forest plot.

#### Anxiety after AC (See supplementary materials)

**Fig. 6:**
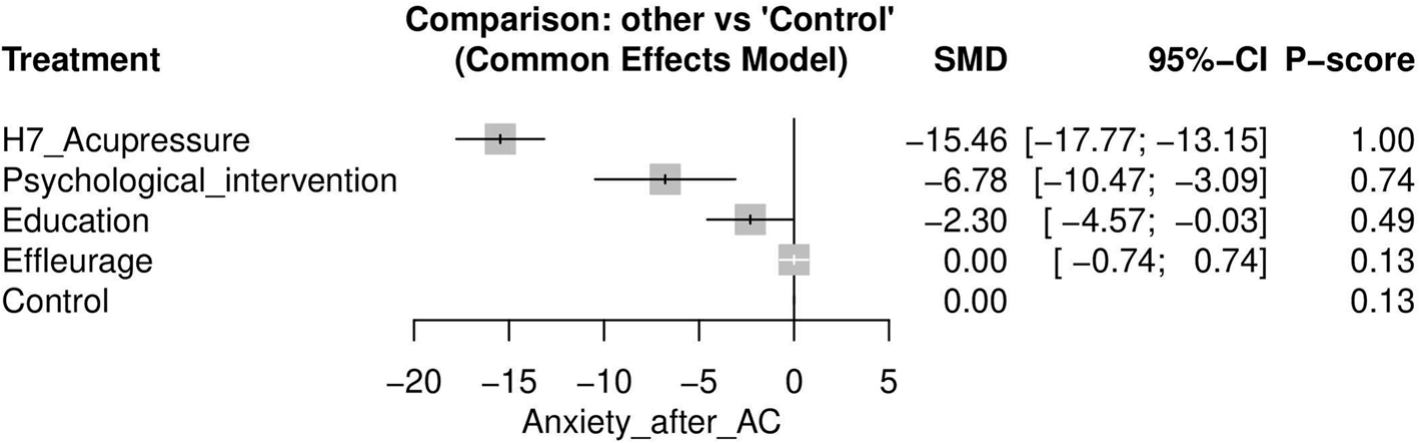
Network meta-analysis forest plot of anxiety after amniocentesis

Anxiety after AC was reported in five studies [16, 29, 33, 36, 37]. Katsogiannou et al. [33], were excluded because their study resulted in two isolated networks. H7-Acupressure was the most effective approach for lowering anxiety after AC when compared to control [SMD = -15.46, 95% CI (-17.77, -13.15)] after which psychological intervention was the next most effective [SMD = -6.78, 95% CI (-10.47, -3.09)]. Figure 6 displays the NMA forest plot.

#### Post-procedure pain and anxiety (See Supplementary materials)

**Fig. 7:**
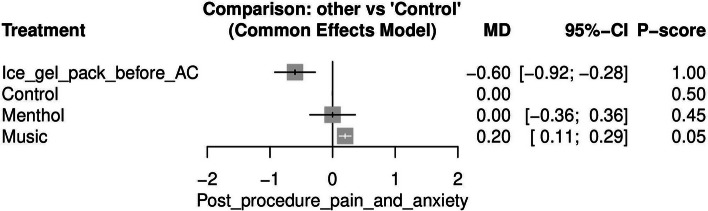
Network meta-analysis forest plot of post-procedure pain and anxiety

Post-procedure pain and anxiety were reported in three studies [20, 31, 32]. The use of Ice gel pack before AC was the most effective method for reducing post-procedure pain and anxiety when compared to the control [MD = -0.60, 95% CI (-0.92, -0.28)]. Figure 7 displays the NMA forest plot.

#### Undergoing AC again if indicated (See Supplementary materials)

**Fig. 8:**
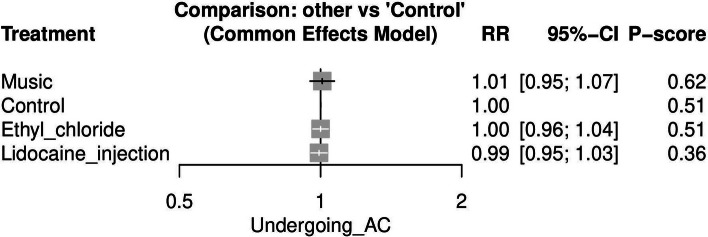
Network meta-analysis forest plot of Undergoing amniocentesis again if indicated

This outcome was reported in three studies [20, 24, 26]. Compared to the control group, the available strategies were not significant. Figure 8 displays the NMA forest plot.

Table 3 Summarizes the findings of the main outcomes.

**Table 3:** Summary of the main findings of the NMA

	**Main outcomes**	**Number of studies included in the NMA**	**The best therapeutic option**	**MD and 95% CI**
**Before the procedure**	Anticipated pain	Ten studies	Ice gel pack	-0.30, (-0.54, -0.06)
	Anxiety before AC	Eight studies	Ice gel pack	-2.30, (-2.43, -2.17)
	Pain during AC	Five studies	Virtual reality	-1.30, (-2.11, -0.49)
**After the procedure**	Pain after AC	14 studies	Paracetamol	-1.68, (-1.99, -1.37)
	*Anxiety after AC*	*Five studies*	*H7 Acupressure*	*-15.46, (-17.77, -13.15)*

*Abbreviations*: *AC* Amniocentesis, *MD* Mean difference, *CI* Confidence interval

## Discussion

The present network meta-analysis compared different strategies to reduce pain and anxiety in prenatal diagnostic procedures. The VR intervention was the most successful in reducing pain during AC. The literature reported that paying attention to pain might affect the pain experience, can be used to explain this. It implies that deflecting attention away from pain can lower perceived pain levels [38]. Distraction is to make a painful stimulus seem less intense since people can’t focus on several sensory inputs at once [39]. Additionally, the VR’s multiple senses may impede nociception flow and lessen pain perception [40]. With a VR headset on, patients can leave the clinical setting and visit another world unconnected to the treatment and any potential pain [41]. Although the exact method by which VR relieves pain is yet unknown, given the well-known connection between pain and emotions, the analgesic impact of VR may be mediated by brain systems [40]. Additionally, VR is simple to use, reasonably priced, and has the benefit of no negative side effects.

The popular pain reliever paracetamol is safe for use during pregnancy [42]. It was successful in lowering pain following AC; however, it was ineffective during AC. The timing of drug intake before the surgery may help to explain this [17]. Further research is needed to determine the ideal premedication period. However, paracetamol has a number of benefits including broad therapeutic applicability, good tolerability, and good absorption after oral administration [43].

The anti-swelling and analgesic effects of cold therapy are widely established for treating soft tissue injuries [44], postoperative pain from gynecologic surgery [45], and perineal pain following vaginal delivery [46]. Reduced soft tissue temperature can have an analgesic effect by slowing the speed at which pain is transmitted. Therefore, applying an ice gel pack before and after receiving AC was useful in lowering pain only after receiving AC, not during receiving AC. Additionally, applying an ice gel pack prior to the AC helped to lower anxiousness. However, a prolonged or excessive exposure might have negative effects like burns and ulcers [46].

H7 Acupressure proved to be successful in lowering anxiety after AC. The results can be explained by a number of various mechanisms. Cortisol and stress are associated in that as stress rises, cortisol also rises. In the hypothalamic–pituitary–adrenal axis, the stimulation of acupressure points alters hormonal-neuronal responses, which in turn controls cortisol output and induces calm. Additionally, by activating the anterior pituitary, acupressure can boost serotonin and dopamine release [47]. Serotonin and dopamine levels in the plasma rise, which reduces cortisol synthesis [48]. In addition, the stimulation of the acupressure point releases endorphins, which peak 30 min after the stimulation begins and remain elevated for 10 h. Similarly, serotonin levels are raised, and endorphins are released when the H7 acupoint is stimulated vigorously and repeatedly [49]. However, other factors must be taken into account while using this method as they may have an impact on the outcomes, such as the target population, the acupressure points employed, and the length of the acupressure.

### Limitations

The majority of the included studies had a high overall risk of bias, which could have an impact on the study’s findings. We exclude some trials to conduct a single network meta-analysis.

## Conclusion

VR was the most effective method for reducing pain during AC, whereas paracetamol was the best method for reducing pain following AC. Additionally, H7 Acupressure was the most effective for reducing anxiety after AC, while an ice gel pack was the best for reducing anxiety before AC. By combining and integrating these methods, healthcare workers will have the potential to significantly aid women who are having AC.

### Supplementary Information

**Additional file 1. **

## Data Availability

The datasets generated during and/or analyzed during the current study are available from the corresponding author upon reasonable request.

## Abbreviations

NMA: Network meta-analysis
AC: Amniocentesis
VR: Visual reality
