# Supplementary material for: Network meta-analysis of randomized controlled trials comparing the effectiveness of different treatments in reducing amniocentesis-associated pain and anxiety
Source: BMC Pregnancy Childbirth. 2023 Nov 21;23:807. doi: 10.1186/s12884-023-06094-3 (PMC10664350; doi:10.1186/s12884-023-06094-3)
Supplement: Supplementary file 1 — Additional file 1. [file 12884_2023_6094_MOESM1_ESM.docx]

**Supplementary Materials**

1. **Risk of bias (ROB) figures**
2. **For each outcome (NMA graph, heterogeneity results, and League table)**
3. **Risk of bias (ROB) figures**

- **Figure S1:** ROB graph

**
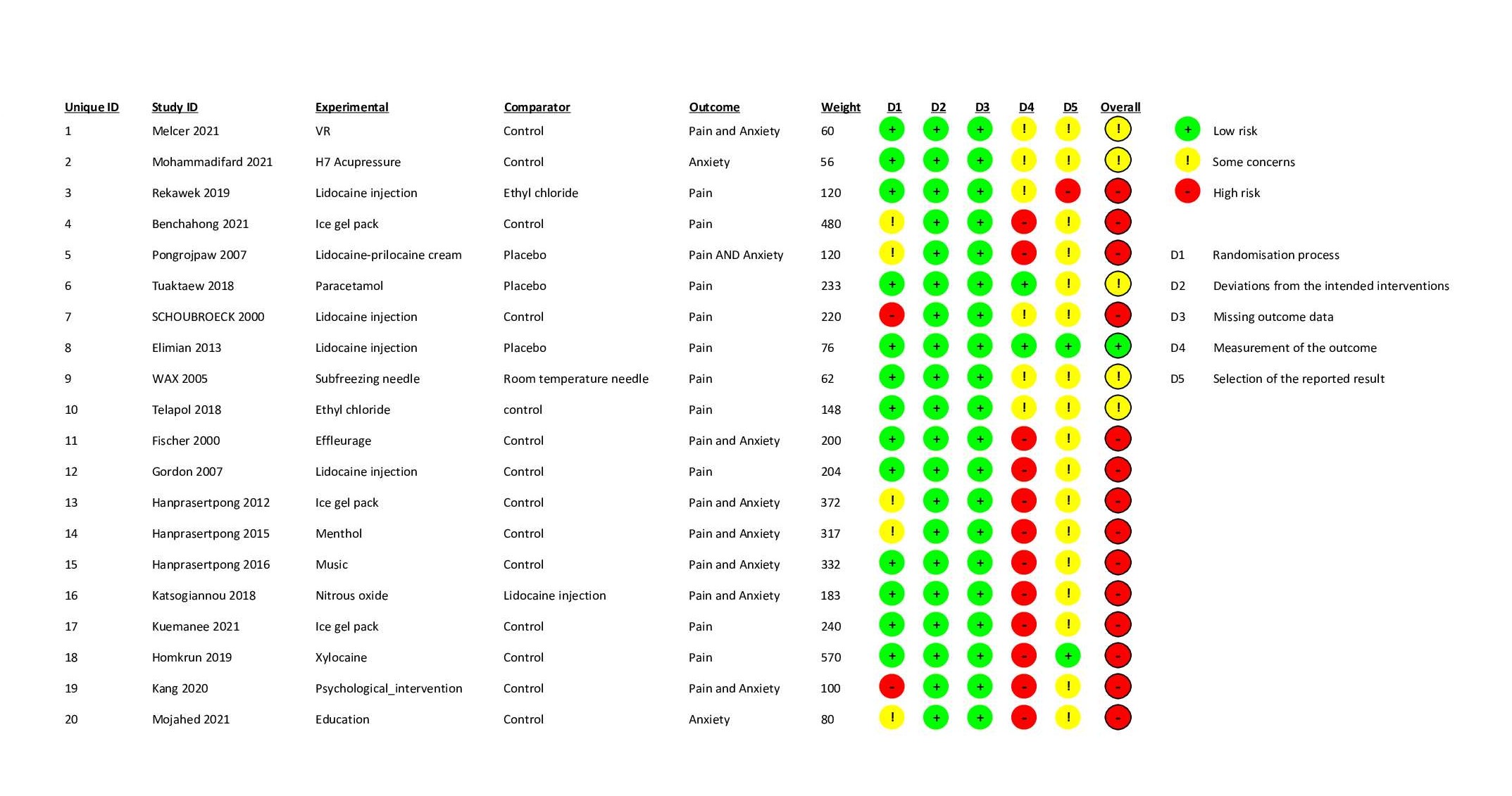
**

- **Figure S2:** ROB Summary

**
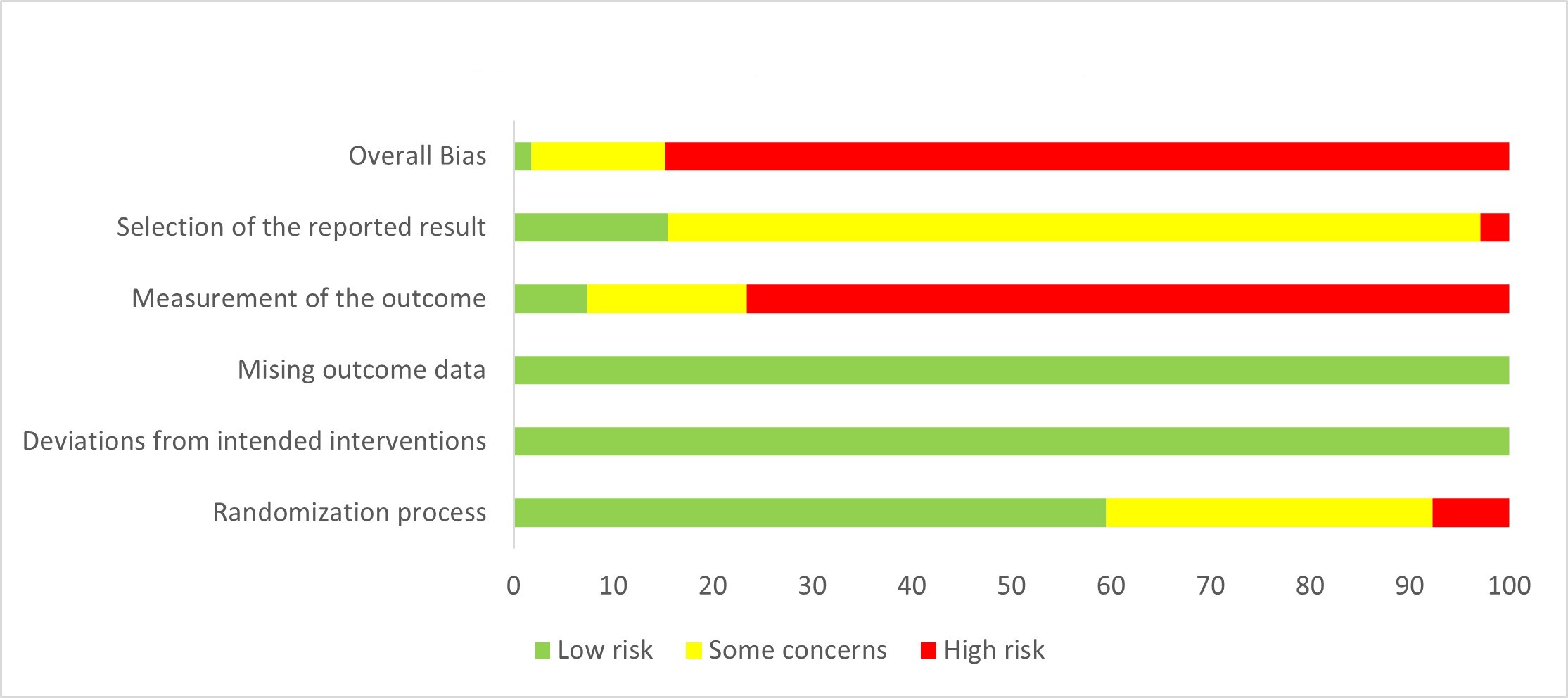
**

1. **For each outcome (NMA graph, heterogeneity results, and League table)**

**1. Anticipated pain**


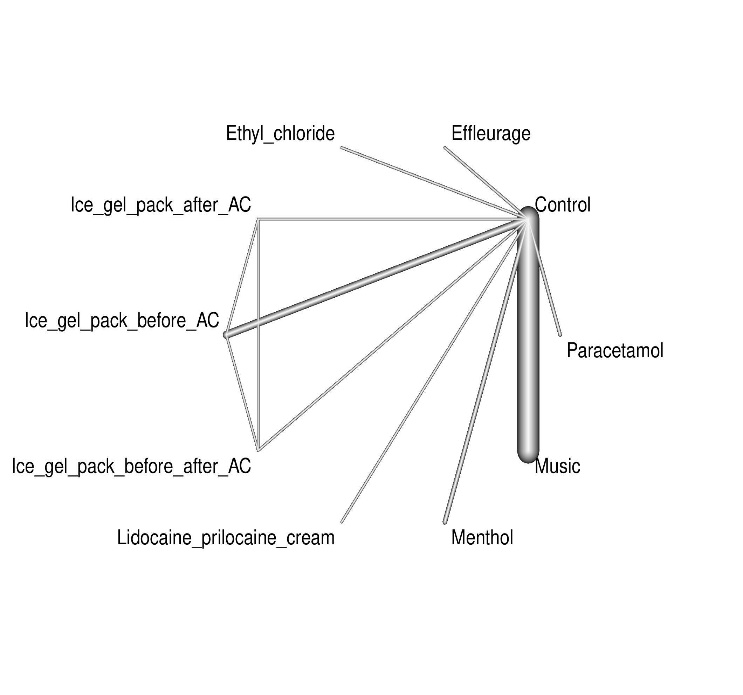


**NMA Graph.**

**Quantifying heterogeneity / inconsistency:**

tau^2 = 0; tau = 0; I^2 = 0% [0.0%; 89.6%]

Tests of heterogeneity (within designs) and inconsistency (between designs):

Q d.f. p-value

Total 1.78 2 0.4111

| Ice_gel_pack_before_AC |  |  |  |  |  |  |  |  |  |
| --- | --- | --- | --- | --- | --- | --- | --- | --- | --- |
| -0.02 [-0.73; 0.69] | Ice_gel_pack_before_after_AC |  |  |  |  |  |  |  |  |
| -0.06 [-0.77; 0.65] | -0.04 [-0.85; 0.77] | Ice_gel_pack_after_AC |  |  |  |  |  |  |  |
| -0.10 [-0.91; 0.71] | -0.08 [-1.13; 0.97] | -0.04 [-1.09; 1.01] | Lidocaine_prilocaine_cream |  |  |  |  |  |  |
| -0.23 [-0.77; 0.32] | -0.21 [-1.07; 0.65] | -0.17 [-1.03; 0.69] | -0.13 [-1.04; 0.78] | Paracetamol |  |  |  |  |  |
| -0.30 [-0.74; 0.14] | -0.28 [-1.08; 0.52] | -0.24 [-1.04; 0.56] | -0.20 [-1.05; 0.65] | -0.07 [-0.68; 0.54] | Menthol |  |  |  |  |
| **-0.30 [-0.54; -0.06]** | -0.28 [-0.99; 0.43] | -0.24 [-0.95; 0.47] | -0.20 [-0.97; 0.57] | -0.07 [-0.56; 0.42] | -0.00 [-0.37; 0.37] | Control |  |  |  |
| -0.55 [-1.33; 0.23] | -0.53 [-1.56; 0.50] | -0.49 [-1.52; 0.54] | -0.45 [-1.52; 0.62] | -0.32 [-1.21; 0.57] | -0.25 [-1.08; 0.58] | -0.25 [-0.99; 0.49] | Ethyl_chloride |  |  |
| -0.60 [-1.29; 0.10] | -0.58 [-1.54; 0.39] | -0.54 [-1.50; 0.43] | -0.50 [-1.51; 0.51] | -0.37 [-1.18; 0.44] | -0.30 [-1.05; 0.45] | -0.30 [-0.95; 0.35] | -0.05 [-1.04; 0.94] | Effleurage |  |
| **-0.60 [-0.85; -0.35]** | -0.58 [-1.29; 0.14] | -0.54 [-1.25; 0.18] | -0.50 [-1.27; 0.27] | -0.37 [-0.86; 0.12] | -0.30 [-0.67; 0.07] | **-0.30 [-0.37; -0.23]** | -0.05 [-0.79; 0.69] | 0.00 [-0.66; 0.66] | Music |

**2. Pain during amniocentesis**


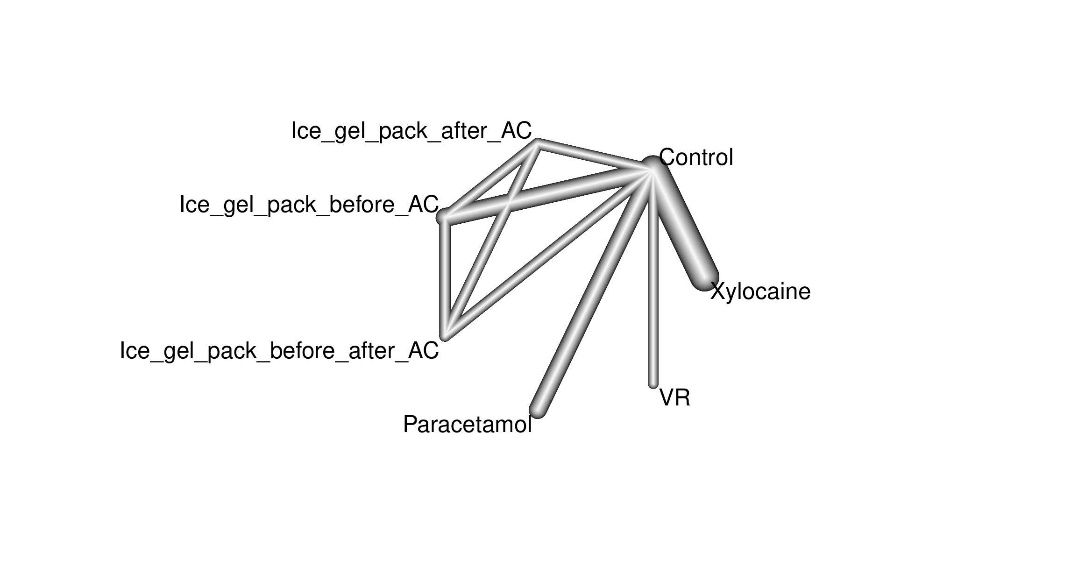


**NMA Graph.**

**Quantifying heterogeneity / inconsistency:**

tau^2 = 0.1278; tau = 0.3575; I^2 = 52.2% [0.0%; 88.0%]

Tests of heterogeneity (within designs) and inconsistency (between designs):

Q d.f. p-value

Total 2.09 1 0.1482

| VR |  |  |  |  |  |  |
| --- | --- | --- | --- | --- | --- | --- |
| -0.37 [-1.44; 0.69] | Ice_gel_pack_before_after_AC |  |  |  |  |  |
| -0.51 [-1.45; 0.43] | -0.14 [-0.98; 0.71] | Paracetamol |  |  |  |  |
| -0.55 [-1.41; 0.31] | -0.18 [-0.93; 0.58] | -0.04 [-0.59; 0.51] | Xylocaine |  |  |  |
| -0.88 [-1.81; 0.05] | -0.50 [-1.20; 0.19] | -0.37 [-1.02; 0.29] | -0.33 [-0.87; 0.21] | Ice_gel_pack_before_AC |  |  |
| **-1.24 [-2.31; -0.18]** | **-0.87 [-1.63; -0.11]** | -0.73 [-1.58; 0.11] | -0.69 [-1.45; 0.06] | -0.37 [-1.06; 0.33] | Ice_gel_pack_after_AC |  |
| **-1.30 [-2.11; -0.49]** | **-0.93 [-1.62; -0.23]** | **-0.79 [-1.26; -0.32]** | **-0.75 [-1.04; -0.46]** | -0.42 [-0.88; 0.03] | -0.06 [-0.75; 0.64] | Control |

**3. Pain after amniocentesis**


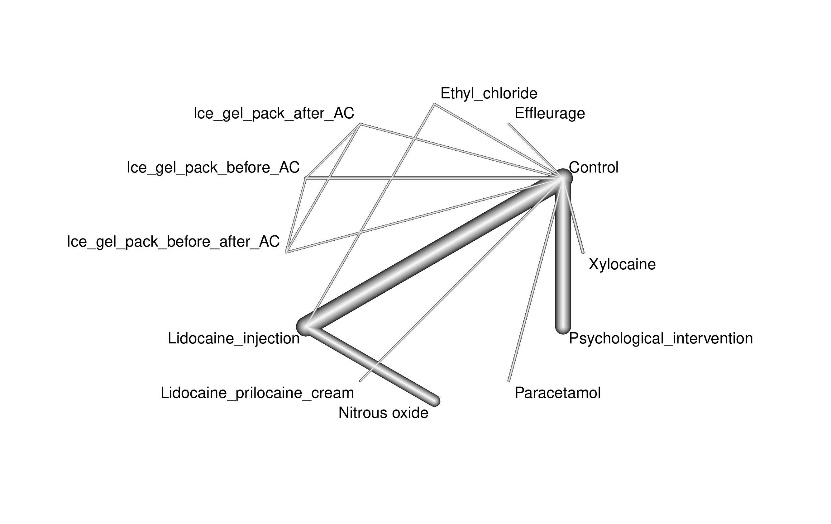


**NMA Graph.**

**Quantifying heterogeneity / inconsistency:**

tau^2 = 0.0028; tau = 0.0528; I^2 = 43.6% [0.0%; 79.3%]

Tests of heterogeneity (within designs) and inconsistency (between designs):

Q d.f. p-value

Total 7.09 4 0.1311

| Paracetamol |  |  |  |  |  |  |  |  |  |  |  |
| --- | --- | --- | --- | --- | --- | --- | --- | --- | --- | --- | --- |
| **-0.77 [-1.27; -0.28]** | Ice_gel_pack_before_after_AC |  |  |  |  |  |  |  |  |  |  |
| **-1.04 [-1.41; -0.67]** | -0.26 [-0.66; 0.13] | Ice_gel_pack_before_AC |  |  |  |  |  |  |  |  |  |
| **-1.11 [-1.80; -0.43]** | -0.34 [-1.07; 0.39] | -0.08 [-0.72; 0.57] | Ethyl_chloride |  |  |  |  |  |  |  |  |
| **-1.08 [-1.98; -0.18]** | -0.31 [-1.23; 0.62] | -0.04 [-0.91; 0.83] | 0.03 [-1.01; 1.07] | Lidocaine_prilocaine_cream |  |  |  |  |  |  |  |
| **-1.25 [-1.75; -0.76]** | **-0.48 [-0.92; -0.04]** | -0.22 [-0.61; 0.18] | -0.14 [-0.87; 0.59] | -0.17 [-1.10; 0.75] | Ice_gel_pack_after_AC |  |  |  |  |  |  |
| **-1.30 [-1.61; -0.98]** | **-0.52 [-0.92; -0.12]** | **-0.26 [-0.48; -0.03]** | -0.18 [-0.80; 0.43] | -0.22 [-1.06; 0.63] | -0.04 [-0.44; 0.36] | Nitrous oxide |  |  |  |  |  |
| **-1.63 [-1.94; -1.32]** | **-0.85 [-1.25; -0.46]** | **-0.59 [-0.80; -0.37]** | -0.51 [-1.12; 0.10] | -0.55 [-1.39; 0.30] | -0.37 [-0.77; 0.02] | **-0.33 [-0.39; -0.27]** | Lidocaine_injection |  |  |  |  |
| **-1.63 [-2.03; -1.23]** | **-0.86 [-1.32; -0.39]** | **-0.59 [-0.93; -0.26]** | -0.52 [-1.18; 0.15] | -0.55 [-1.43; 0.33] | -0.38 [-0.84; 0.09] | **-0.33 [-0.60; -0.06]** | -0.00 [-0.26; 0.26] | Xylocaine |  |  |  |
| **-1.67 [-1.98; -1.36]** | **-0.90 [-1.29; -0.50]** | **-0.63 [-0.85; -0.41]** | -0.56 [-1.17; 0.06] | -0.59 [-1.43; 0.25] | **-0.42 [-0.81; -0.02]** | **-0.37 [-0.46; -0.29]** | -0.04 [-0.10; 0.02] | -0.04 [-0.30; 0.22] | Psychological_intervention |  |  |
| **-1.68 [-1.99; -1.37]** | **-0.91 [-1.30; -0.51]** | **-0.64 [-0.85; -0.43]** | -0.57 [-1.18; 0.04] | -0.60 [-1.44; 0.24] | **-0.43 [-0.82; -0.03]** | **-0.38 [-0.46; -0.31]** | **-0.05 [-0.09; -0.01]** | -0.05 [-0.31; 0.21] | -0.01 [-0.06; 0.04] | Control |  |
| **-1.98 [-2.70; -1.26]** | **-1.21 [-1.97; -0.45]** | **-0.94 [-1.63; -0.26]** | -0.87 [-1.76; 0.03] | -0.90 [-1.97; 0.17] | -0.73 [-1.49; 0.03] | **-0.68 [-1.34; -0.03]** | -0.35 [-1.01; 0.30] | -0.35 [-1.05; 0.35] | -0.31 [-0.96; 0.34] | -0.30 [-0.95; 0.35] | Effleurage |

**4. Anxiety before amniocentesis**


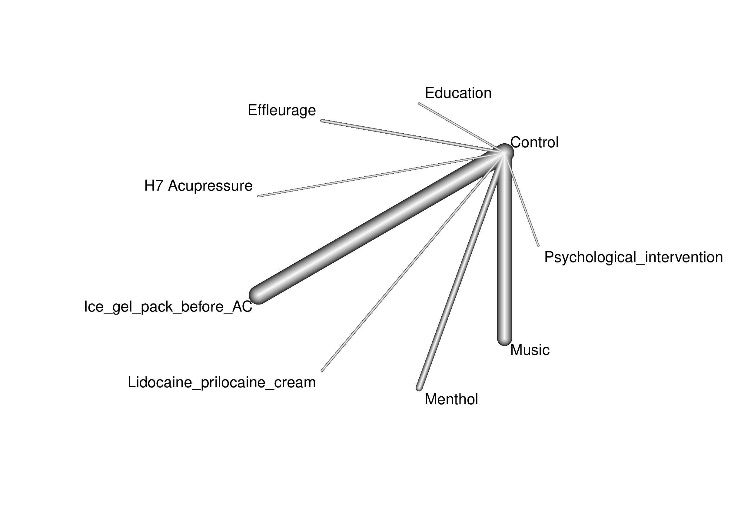


**NMA Graph.**

**Quantifying heterogeneity / inconsistency:**

tau^2 = NA; tau = NA

Tests of heterogeneity (within designs) and inconsistency (between designs):

Q d.f. p-value

Total 0 0 --

| Ice_gel_pack_before_AC |  |  |  |  |  |  |  |  |
| --- | --- | --- | --- | --- | --- | --- | --- | --- |
| -0.30 [-0.69; 0.09] | Menthol |  |  |  |  |  |  |  |
| **-2.10 [-3.38; -0.82]** | **-1.80 [-3.13; -0.47]** | H7 Acupressure |  |  |  |  |  |  |
| -2.07 [-5.32; 1.18] | -1.77 [-5.04; 1.50] | 0.03 [-3.46; 3.52] | Psychological_intervention |  |  |  |  |  |
| **-2.30 [-2.43; -2.17]** | **-2.00 [-2.37; -1.63]** | -0.20 [-1.48; 1.08] | -0.23 [-3.48; 3.02] | Control |  |  |  |  |
| **-2.50 [-3.34; -1.66]** | **-2.20 [-3.11; -1.29]** | -0.40 [-1.93; 1.13] | -0.43 [-3.79; 2.93] | -0.20 [-1.03; 0.63] | Effleurage |  |  |  |
| **-2.60 [-3.74; -1.46]** | **-2.30 [-3.49; -1.11]** | -0.50 [-2.21; 1.21] | -0.53 [-3.97; 2.91] | -0.30 [-1.43; 0.83] | -0.10 [-1.50; 1.30] | Lidocaine_prilocaine_cream |  |  |
| **-3.10 [-3.31; -2.89]** | **-2.80 [-3.20; -2.40]** | -1.00 [-2.29; 0.29] | -1.03 [-4.29; 2.23] | **-0.80 [-0.97; -0.63]** | -0.60 [-1.45; 0.25] | -0.50 [-1.64; 0.64] | Music |  |
| **-4.20 [-7.55; -0.85]** | **-3.90 [-7.27; -0.53]** | -2.10 [-5.68; 1.48] | -2.13 [-6.80; 2.54] | -1.90 [-5.25; 1.45] | -1.70 [-5.15; 1.75] | -1.60 [-5.13; 1.93] | -1.10 [-4.45; 2.25] | Education |

**5. Anxiety after amniocentesis**


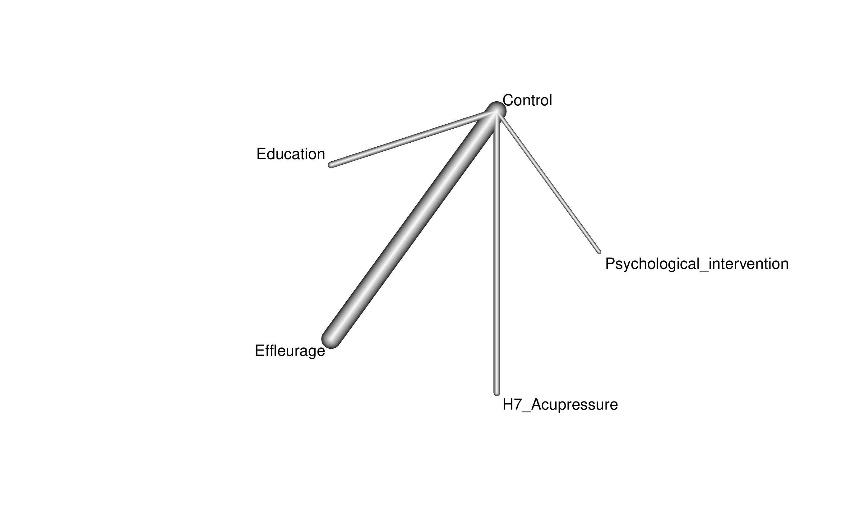


**NMA Graph.**

**Quantifying heterogeneity / inconsistency:**

tau^2 = NA; tau = NA

Tests of heterogeneity (within designs) and inconsistency (between designs):

Q d.f. p-value

Total 0 0 --

| H7_Acupressure |  |  |  |  |
| --- | --- | --- | --- | --- |
| **-8.68 [-13.04; -4.32]** | Psychological_intervention |  |  |  |
| **-13.16 [-16.40; -9.92]** | **-4.48 [ -8.81; -0.15]** | Education |  |  |
| **-15.46 [-17.89; -13.03]** | **-6.78 [-10.55; -3.01]** | -2.30 [ -4.69; 0.09] | Effleurage |  |
| **-15.46 [-17.77; -13.15]** | **-6.78 [-10.47; -3.09]** | **-2.30 [ -4.57; -0.03]** | 0.00 [ -0.74; 0.74] | Control |

**6. Post procedure pain and anxiety**


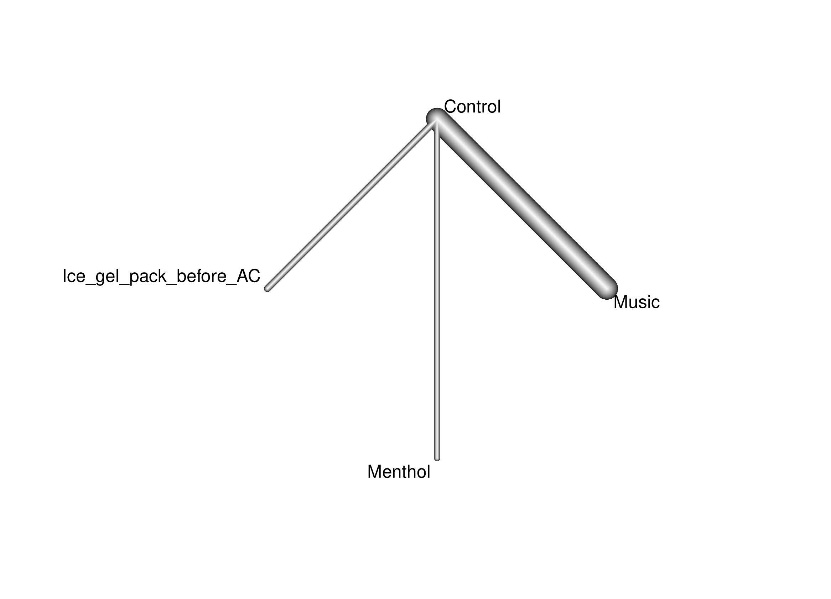


**NMA Graph.**

**Quantifying heterogeneity / inconsistency:**

tau^2 = NA; tau = NA

Tests of heterogeneity (within designs) and inconsistency (between designs):

Q d.f. p-value

Total 0 0 --

| Ice_gel_pack_before_AC |  |  |  |
| --- | --- | --- | --- |
| **-0.60 [-0.92; -0.28]** | Control |  |  |
| **-0.60 [-1.09; -0.11]** | -0.00 [-0.36; 0.36] | Menthol |  |
| **-0.80 [-1.14; -0.46]** | **-0.20 [-0.29; -0.11]** | -0.20 [-0.57; 0.17] | Music |

**7. Undergoing amniocentesis again if indicated**


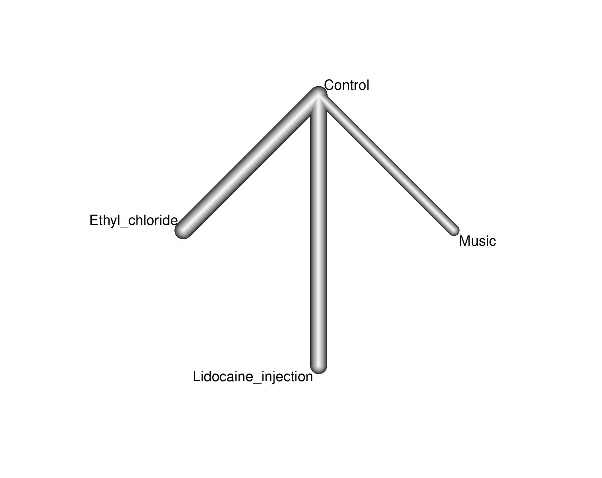


**NMA Graph.**

**Quantifying heterogeneity / inconsistency:**

tau^2 = NA; tau = NA

Tests of heterogeneity (within designs) and inconsistency (between designs):

Q d.f. p-value

Total 0 0 --

| Music |  |  |  |
| --- | --- | --- | --- |
| 1.01 [0.95; 1.07] | Control |  |  |
| 1.01 [0.94; 1.08] | 1.00 [0.96; 1.04] | Ethyl_chloride |  |
| 1.02 [0.95; 1.09] | 1.01 [0.97; 1.05] | 1.01 [0.95; 1.06] | Lidocaine_injection |
